# Promising Nanocarriers for PEDF Gene Targeting Delivery to Cervical Cancer Cells Mediated by the Over-expressing FRα

**DOI:** 10.1038/srep32427

**Published:** 2016-08-31

**Authors:** Yuhan Yang, Lili He, Yongmei Liu, Shan Xia, Aiping Fang, Yafei Xie, Li Gan, Zhiyao He, Xiaoyue Tan, Chunling Jiang, Aiping Tong, Xiangrong Song

**Affiliations:** 1 State Key Laboratory of Biotherapy and Cancer Center, West China Hospital, Sichuan University, and Collaborative Innovation Center for Biotherapy, Sichuan University, Chengdu 610041, China; 2 College of Pharmacy, Southwest University for Nationalities, Chengdu 610041, China; 3 Chemistry and Life Science College, Chengdu Normal University, Chengdu 611130, China; 4 Department of Pathology/Collaborative Innovation Center of Biotherapy, Medical School of Nankai University, Tianjin 300071, China

## Abstract

Cervical cancer presents extremely low PEDF expression which is associated with tumor progression and poor prognosis. In this study, folate receptor α (FRα)-targeted nano-liposomes (FLP) were designed to enhance the anti-tumor effect by targeting delivery of exogenous PEDF gene to cervical cancer cells. The targeting molecule F-PEG-Chol was firstly synthesized by a novel simpler method. FLP encapsulating PEDF gene (FLP/PEDF) with a typical lipid-membrane structure were prepared by a film dispersion method. The transfection experiment found FLP could effectively transfect human cervical cancer cells (HeLa cells). FLP/PEDF significantly inhibited the growth of HeLa cells and human umbilical vein endothelial cells (HUVEC cells) and suppressed adhension, invasion and migration of HeLa cells *in vitro*. In the abdominal metastatic tumor model of cervical cancer, FLP/PEDF administered by intraperitoneal injection exhibited a superior anti-tumor effect probably due to the up-regulated PEDF. FLP/PEDF could not only sharply reduce the microvessel density but also dramatically inhibit proliferation and markedly induce apoptosis of tumor cells *in vivo*. Moreover, the preliminary safety investigation revealed that FLP/PEDF had no obvious toxicity. These results clearly showed that FLP were desired carriers for PEDF gene and FLP/PEDF might represent a potential novel strategy for gene therapy of cervical cancer.

Cervical cancer, also called invasive cervical carcinoma, is the second malignant tumor to women all over the world^1^. The early cervical cancer is absent of symptoms and could be curable by surgical intervention, radiation treatment and chemotherapy^2^, among which radiation treatment and chemotherapy could produce serious side effect harming normal tissues. Moreover, the advanced and/or metastatic or recurrent tumors are the deadly ones to those patients with poor prognosis and less than 5% of them could be alive at 5 years^3^. Fortunately, gene therapy-based therapeutics in clinical trials, including growth factors, tumor suppressors, antigens, receptors and others^4^, have offered many exciting possibilities for treating cervical cancer.

Pigment epithelium-derived factor (PEDF) is an endogenous 50 kDa glycoprotein disseminating throughout the body^5^, which has been proved to present not only direct anti-tumor properties of apoptosis^6^, differentiation and anti-proliferation^7^ of tumor cells but also indirect characters like anti-angiogenesis^8^ possibly through vascular endothelial growth factor (VEGF) and inducing apoptosis of endothelial cells. Actually, PEDF has been reported to be as an anti-cancer agent against a range of tumors such as lung, breast, prostate, and ovary^9^,^10^,^11^,^12^,^13^, but rarely in cervix. PEDF gene therapy might be a more promising therapeutic strategy in contrast to PEDF itself owing to more lasting curative effect, easier scale-up production and lower cost. PEDF gene indeed had better therapeutic efficacy on many tumors such as melanoma, hepatocellular carcinoma and colon carcinoma^14^,^15^,^16^. Nevertheless, there was still no report about PEDF gene therapy for cervix cancer, probably lacking a specific and efficient delivery carrier.

We previously developed a series of nanocarriers for PEDF gene delivery based on PLGA nanoparticles, which had achieved better *in vivo* therapeutic efficacy for colon carcinoma^17^,^18^. However, these vectors had no active selectivity for cervix cancer cells. Cervical tumor tissues over-express folate receptor α (FRα) especially in metastatic focus and recurrent tumors^19^, which could provide conditions for folate-mediated targeting delivery for PEDF gene. Folate-decorated nanoparticles have been reported to successfully targeting deliver low molecular weight chemotherapeutic agents^20^ or theranostic agents^21^ to cervix cancer tissues *in vivo.* Our group formerly constructed a novel non-viral gene vector, folate-modified nano-liposomes (FLP) which could specifically deliver therapeutic gene CLDN3 shRNA to ovarian cancer cells and thereby achieve high *in vivo* anti-tumor efficacy by recognizing the up-regulated FRα^22^. Therefore, we hypothesized that FLP might also be suitable nano-carrieres for efficient delivery of PEDF gene to cervical tumor cells.

In the present study, we investigated whether FLP could be used as PEDF gene vectors to battle cervix cancer. The targeting molecule F-PEG-Chol was firstly synthesized and purified by a novel simpler method. FLP, prepared by a self-assembly process, were subsequently complexed with PEDF gene to form FRα-targeted nano-lipoplexes (FLP/PEDF). Finally, FLP/PEDF were injected intraperitoneally to treat the peritoneal metastasis of cervical cancer. To our knowledge, we show for the first time that FLP are highly effective in delivering PEDF gene for cervix cancer therapy.

## Experimental Section

### Materials

1,2-dioleoyl-3-trimethylammonium-propane (DOTAP) was purchased from Avanti Polar Lipids Inc. (Alabaster, AL, USA). Cholesterol (Chol) was obtained from Chengdu Kelong Chemical Co. Ltd & ChengDu Kelong Chemical Reagent Company (Chengdu, China). Cholesterol succinic anhydride ester (Chol-suc) was synthesized as described previously^23^. Poly (ethylene glycol) diamine (H_2_N-PEG-NH_2_) was provided by BioMatrik Inc. (Jiaxing, Zhejiang, China). MPEG-succinyl-Cholesterolconjugate (mPEG-Chol) was synthesized and purified by our research group^24^,^25^. The plasmid green fluorescent protein (pGFP), PEDF plasmid PAAV_2_-PEDF (PEDF gene) and null plasmid PAAV_2_ were constructed according to our previous report^16^. Plasmid DNA (pDNA) was extracted according to the EndoFree Plasmid Purification Handbook (QIAGEN, Germany). DNA ladder and pre-stained protein ladder were provided by Fermentas (Thermo Fisher Scientific Inc., Waltham, MA, USA). All reagents were of analytical grade and were used without further purification except chloroform used to prepare nano-liposomes.

### Synthesis and Characterization of F-PEG-Chol

**Figure 1:**
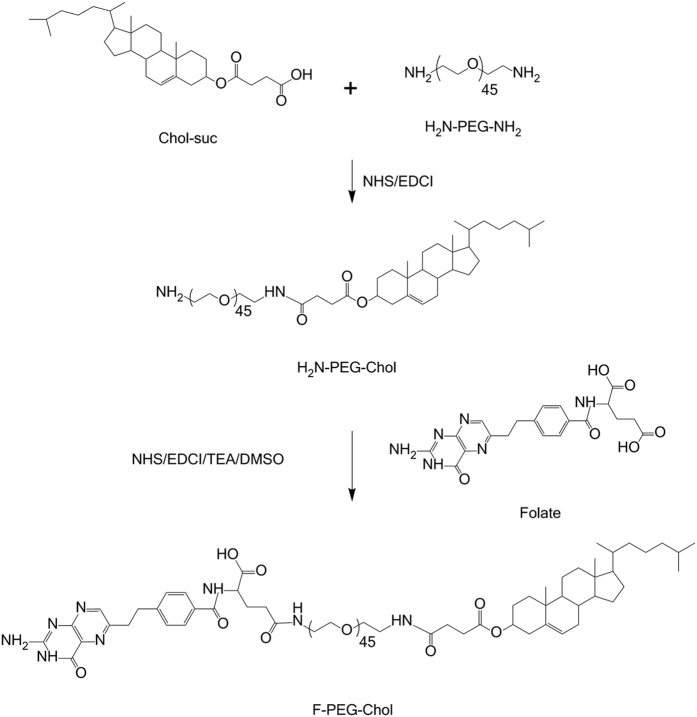
The synthesis route of F-PEG-Chol. F-PEG-Chol was synthesized through a simple two-step reaction. Specifically, Chol-suc and H_2_N-PEG-NH_2_ were used to prepare the intermediate H_2_N-PEG-Chol which was subsequently reacted with folate to form F-PEG-Chol.

The synthesis route of F-PEG-Chol was presented in Fig. 1. According to our previous report^26^, F-PEG-Chol was synthesized through a simple two-step reaction. Briefly, Chol-suc and H_2_N-PEG-NH_2_ were used to prepare the intermediate H_2_N-PEG-Chol which was subsequently reacted with folate to form F-PEG-Chol. In this study, the synthetic process parameters and the purified method of H_2_N-PEG-Chol were further optimized. Specifically, the mixture containing Chol-suc, NHS and EDCI (1:4:2, molar ratio) in dichloromethane reacted to generate the activated ester, which was then dropped into H_2_N-PEG-NH_2_ (molar ratio to mixture was 1.2). The reaction mixture was stirred at room temperature and monitored at the same time. Once Chol-PEG-Chol was detected, the reaction was terminated. The reaction liquid was washed by 1 M HCl solution and then saturated NaHCO_3_ solution. The organic layer was dried with Na_2_SO_4_ and then rotarily evaporated. The obtained crude product was re-dissolved with dichloromethane, followed by recrystallization in petroleum ether and then centrifuged to get white crystal. After washed by diethyl ether, the product H_2_N-PEG-Chol was air-dried. ^1^H-NMR spectra of H_2_N-PEG-Chol, Chol-suc and H_2_N-PEG-NH_2_ in CDCl_3_ were recorded on a Unity Inova-400 (400 MHz) (Varian Inc., Palo Alto, CA, USA) at room temperature.

### Preparation and Characterization of FLP/PEDF

FLP were prepared by a film dispersion method as described previously^25^. In detail, DOTAP, Chol, mPEG-Chol and F-PEG-Chol were dissolved in chloroform. The solution was rotarily evaporated to remove the solvent and then the make-up film was further dried under high vacuum for 4–6 h. The dry lipid film was hydrated in 5% glucose solution, and the obtained suspension of lipids were sonicated by a probe to form translucent liposome solution. Thereafter, the colloidal solution was sterilized through a Millipore 0.22 μm filter membrane to obtain FLP and stored at 4 °C before used. This method was also used to construct non-targeted nano-liposomes (PLP) containing DOTAP, Chol and mPEG-Chol and normal nano-liposomes (LP) consisting of DOTAP and Chol.

FLP/PEDF were prepared by mixing FLP with PEDF gene. Briefly, 1 mg/mL FLP in 5% glucose solution was added to 1 mg/mL PEDF in TE buffer. The mixture was mixed slightly and quickly for 3 times. After incubating for 30 min at room temperature, FLP/PEDF was finally formed. FLP/PAAV_2_ were produced by mixing FLP with PAAV_2_ in the same procedure. PLP/PEDF and PLP/PAAV_2_ were assembled by the same approach as FLP/PEDF and FLP/PAAV_2_, respectively. The particle size and zeta-potential of FLP/PEDF were measured by Zetasizer NanoZS ZEN 3600 (Malvern Instruments, Ltd., Malvern, Worcestershire, UK). The morphological characteristic was examined by a transmission electron microscope (TEM, H-600, Hitachi, Ltd, Japan).

Agarose gel electrophoresis was used to test the gene loading capacity of FLP. The electrophoresis was proceeded on 1% (w/v) agarose gel (Invitrogen Corp., Carlsbad, USA) in pH 7.4 TAE buffer containing Golden View as nucleic acid stain. 10 μL of FLP/PEDF mixed with 2 μL of loading buffer (Thermo Fisher Scientific Inc, USA) were applied to agarose at a constant voltage of 130 V for 20 min at room temperature. The electrophoresis gel was visualized and digitally photographed by a gel documentation system (Gel Doc 1000, Bio-Rad Laboratories, Hercules, CA, USA).

### *In vitro* Anti-Tumor Activity Study

#### Cell Culture

Human cervical cancer cells (HeLa cells) were obtained from the American Type Culture Collection. The cells were cultured as a monolayer in Roswell Park Memorial Institute-1640 Medium (RPMI1640, Gibco, Invitrogen Corp., Carlsbad, CA, USA) extra added with 10% calf serum, L-glutamine (2 mM), penicillin (100 U/mL) and streptomycin (100 μg/mL). Human umbilical vein endothelial cells (HUVEC cells) were obtained from the newborn’s umbilical cord. The cells were cultured as a monolayer in Dulbecco’s Modified Eagle’s Medium (DMEM, Gibco, Invitrogen Corp., Carlsbad, CA, USA) supplemented with 10% fetal bovine serum and others were the same as above. They were both cultured in a humidified atmosphere containing 5% CO_2_ at 37 °C.

#### Detection of FRα Expressed on HeLa cells

HeLa cells in culture dish were harvested after trypsinization with 0.25% EDTA and centrifuged, and the cells sediments were washed with PBS. They were divided into four groups as negative, second antibody, isotype control and positive group. 2 μL anti-hFOLR1 purified mouse monoclonal IgG1 (R&D Systems, Minneapolis, MN, USA) was added to the re-suspended cells in PBS in the isotype control and positive group. After incubation for 2 h at 4 °C, they were washed with PBS and then centrifuged, and finally resuspended with PBS. 2 μL anti-mouse IgG (Fc specific)-FITC (Sigma-Aldrich, St. Louis, MO, USA) was added to the second antibody group and positive group. After incubation for 30 min at 4 °C, the resuspended cell suspensions in PBS were analyzed by flow cytometer (BD Biosciences, San Jose, CA, USA) to determine the positive rate.

#### Transfection experiment

Transfection experiment was done as described previously^25^. Lipo2000/GFP were prepared according to the protocol of Lipofectamine™ 2000 (Lipo2000). FLP/GFP, PLP/GFP and LP/GFP were formulated by mixing GFP with FLP, PLP and LP in the same approach as FLP/PEDF, respectively. HeLa cells were seeded on 6-well plates (Corning Inc., NY, USA) at 5 × 10^4^ cells per well in 2 mL RPMI1640 with 10% calf serum. After adherence for 24 h, the culture medium was replaced by 800 μL of folate free RPMI1640 (serum-free) in each well. The nano-lipoplexes (FLP/GFP, PLP/GFP, LP/GFP and Lipo2000/GFP) in a final volume of 200 μLcontaining 1 μg GFP gene were subsequently added to the wells. After 6 h of incubation, cell culture medium was changed to RPMI1640 with calf serum and the cells were incubated for extra 42 h. The expressed GFP was observed under an Olympus IX 71 inverted fluorescence microscope (Olympus Corp., Tokyo, Japan). Then the cells were harvested after trypsinization with 0.25% EDTA and centrifuged, and the cells sediments were washed with PBS. The re-suspended cell suspensions in PBS were analyzed by flow cytometer to determine the transfection efficiency.

#### Cytotoxicity assay of FLP/PEDF

Cytotoxicity of FLP/PEDF was carried out by MTT assay on HeLa cells and HUVEC cells as described previously^27^. In brief, cells were planted into 96-well plates (Corning Inc., NY, USA) at a density of 3 × 10 ^3^ cells per well in 100 μL medium. After attaching overnight, cells were treated with another 100 μL various concentrations of FLP/PEDF and further incubated for another 24 h, 48 h or 72 h to assess concentration-dependent and time-dependent cytotoxicity. At the end of predetermined time, 20 μL MTT solution (5 mg/mL in saline) was added to each well and cells were further cultured at 37 °C for extra 4 h. Finally, the culture media were removed and then 150 μL DMSO was added to each well to dissolve formazan crystals. The OD value of each well was read at 490 nm on a Multiskan MK3 microplate reader (Thermo Fisher Scientific Inc., Waltham, MA, USA). The parallel cytotoxicity experiment was performed on the contrast formulations including FLP/PAAV_2_, PLP/PEDF, PLP/PAAV_2_, FLP, PLP and Lipo2000 in parallel experiment. Untreated cells were used as controls. The relative cell viability compared to control was calculated as below:


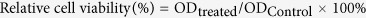


### *In vitro* Anti-metastasis Research

#### Adhesion assay

Adhesion assay was operated by MTT assay^28^. HeLa cells were firstly transfected with PLP/PAAV_2_, FLP/PAAV_2_, PLP/PEDF and FLP/PEDF, respectively. Briefly, HeLa cells were seeded on 6-well plates at 3 × 10^5^ cells per well in 2 mL RPMI1640 with 10% calf serum. After adherence for 24 h, the culture medium was replaced by 800 μL of folate free RPMI1640 in each well. The nano-lipoplexes containing 5 μg PEDF gene or PAAV_2_ in 200 μL folate free RPMI1640 were subsequently added to the wells. HeLa cells transfected with nano-lipoplexes were finally collected after co-culture for 48 h. The treated cells were used as the experimental groups while the non-transfected HeLa cells were the control group. All the cells were planted into the Matrigel-coated 96-well plate in triplicate. After attachment for the predetermined time (30 min, 60 min and 90 min), the plate was washed to remove the non-adherent cells. The amount of attached cells was then measured by MTT assay at 490-nm wavelength. The relative adhesive rate compared to control was calculated as below:


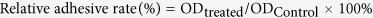


#### Invasion assay

The invasion ability was investigated by transwell assay^29^. 200 μL HeLa cells (2 × 10^5^/mL in serum free RPMI-1640) transfected with nano-lipoplexes (PLP/PAAV_2_, FLP/PAAV_2_, PLP/PEDF and FLP/PEDF) were placed on the top chamber of each transwell (8 μm pore size, Corning, America) coated with 50 μL diluted Matrigel (volume ratio to RPMI-1640 serum-free was 1:3). The bottom chamber was filled with 500 μL RPMI1640 medium with 10% calf serum to act as the nutritional attractant. At the end of invasion for 24 h, non-migrated cells on the top surface of membrane were removed by cotton swabs and then washed with PBS. Migrated cells on the bottom surface of membrane were fixed, stained, and counted. The migrated cells were observed under an Olympus BX 53 fluorescence microscope (Olympus Corp., Tokyo, Japan). All the experiments were performed independently in triplicate.

#### Scratch assay

HeLa cells were firstly planted into 6-well plates. When cells spread to 80–90%, straight lines were drawn by a pipette tip on the bottom of plate. The cells were then washed with PBS to remove the non-adhered cells. The nano-lipoplexes mentioned above were added to transfect all the cells. After 48 h, the cell migration into the scratches was observed under Olympus IX 71 inverted fluorescence microscope. The migration distance ratio was calculated as below^30^:


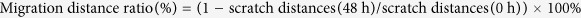


### *In vivo* Anti-cancer Study

#### Animals

Female athymic nude mice (BALB/c-nude, SPF) were purchased from Vital River (Beijing, China). The mice were fed under SPF conditions. All studies were approved and supervised by the State Key Laboratory of Biotherapy Animal Care and Use Committee (Sichuan University, Chengdu, Sichuan, China) and conform to the Guide for the Care and Use of Laboratory Animals published by the US National Institutes of Health. The mice were used until they were 6 to 8 weeks old.

#### Heterotopic Tumor Growth Assay

Mice were injected intraperitoneally with 0.2 mL HeLa cells suspension (1 × 10^7^ cells in serum free RPMI-1640) and randomly separated into five groups based on their body weights two days after inoculation. They were administered once every third day with the nano-lipoplexes containing plasmid DNA (5 μg) in 200 μL volume. Treatment continued until the mice of the control group became moribund (typically 4 to 6 weeks). At the time of sacrifice, tumor tissues, whole blood, ascitic fluid and organs of mice were harvested. The ascitic fluid volumes, tumor weights and numbers of nodules were recorded. The tumor specimens were divided into two portions: Parts of the tumors were immediately lysed by RIPA lysis buffer (Sigma-Aldrich, St. Louis, MO, USA) containing proteinase inhibitor cocktail (Sigma-Aldrich, St. Louis, MO, USA), centrifuged, and the supernatant was stored at −80 °C for western blot analysis. The rest tumor tissues were fixed with paraformaldehyde in PBS (pH 7.4) and then imbedded by paraffin for tissue sectioning.

#### Immunohistochemistry Staining

Labeled streptavidin-biotin method was used in immunohistochemistry (IHC). PEDF expression, Ki_67_ antigen and microvessel density (MVD, CD31) were analyzed with human serpin F1/PEDF antibody (R&D, USA), rabbit anti-human Ki_67_ antibody (AbcamPLC, Boston, MA, USA) and rabbit anti-human CD31 antibody (AbcamPLC, Boston,MA, USA). Briefly, 4–5 μm sections were made from paraffin-embedded tumor tissue specimens of each group and subsequently deparaffinized by sequentially washed with xylene (I and II), 100% ethanol, 95% ethanol, 85% ethanol, 75% ethanol and water. Endogenous peroxide was blocked with 3% H_2_O_2_ kept in dark place at room temperature for 10 min. Antigen retrieval was done by heated in a pressure cooker in 10 mM sodium citrate buffer (pH 6.0). After washed with PBS, tissues were blocked with goat serum for 30 min at 37 °C then incubated with primary antibody overnight at 4 °C. After washed with PBS for three times, the secondary antibody conjugated to horse radish peroxidase (HRP) was added. HRP was detected with 3,3′-diaminobenzidine substrate (DAB Kit, ZSGB-Bio, Beijing, China) for 30 s or more, terminated by water and re-dyed with hematoxylin (Beyotime Institute of Biotechnology, Shanghai, China) for 30–60 s. 10 random fields at ×400 magnification were examined for each section.

#### TUNEL Assay

Apoptotic cells were detected on paraffin sections according to the protocol of terminal deoxynucleotidyl transferase-mediated nick-end labeling (TUNEL) staining. Briefly, the sections mentioned above were deparaffinized by the same process as IHC. After washed with 0.85% sodium chloride solution and PBS and fixed with paraformaldehyde, sections were incubated with proteinase K solution (20 μg/mL) and then rTDT buffer at 37 °C for 60 min in a dark place. The sections were observed under a fluorescence microscope and digitally photographed by the Olympus Application Suite (Olympus Corporation, Japan). Cells with dark green fluorescent staining were defined as TUNEL-positive cells while total number of cells were counted in 10 random fields at ×400 magnification. Percent apoptosis was determined as below:


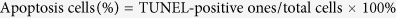


#### Western Bolt Analysis

Total protein concentrations of tumor tissues lysates were measured through the Bradford protein assay reagent kit (Bio-Rad Laboratories, Hercules, CA, USA). The PEDF protein was separated by 10% SDS-PAGE under reducing conditions and then transferred to Millipore PVDF membranes. Membranes were blocked with 5% skimmed milk and incubated with anti-PEDF antibody (R&D Systems, Minneapolis, MN, USA) at 4 °C overnight. Antibodies were detected with horseradish peroxidase (HRP)-conjugated secondary antibody and developed with an enhanced chemiluminescence detection kit (Luminata Crescendo Western HRP Substrate, or Immobilon Western Chemiluminescent HRP Substrate, Millipore Corporation, Billerica, MA, USA). Membranes were tested for β-actin (Santa Cruz Biotechnology, Inc., Santa Cruz, CA, USA) to confirm equal loading.

#### ELISA Analysis

After serum was acquired as above, ELISA analysis was proceeded according to the protocol of human pigment epithelium-derived factor (PEDF) ELISA KIT (R&D Systems, Minneapolis, MN, USA). In brief, samples were added into a monoclonal antibody (McAb)-coated pore plate to incubate for 30 min at 37 °C. After washed for 5 times, enzyme standard reagent was added for 50 μL per well following incubated and washed. After added A solution, B solution and stop buffer in turn, samples were measured with MTT assay at 450-nm wavelength.

### Safety Evaluation

#### Body Weight, Continuously Observation and HE Staining

To estimate the potential side effects in the FLP/PEDF-treated mice, they were continuously observed for appearance, weight, independent activity and toxic deaths. The body weights were measured and recorded once three days. Vital organs (heart, liver, spleen, lung and kidney) were collected after sacrifice for HE staining and observed by two pathologists in a blinded manner.

#### Serological and Biochemical Analysis

Whole blood, obtained from mice, was divided into two parts. One was directly used to conduct a whole blood analysis using a Celltac alpha MEK-6318K fully automatic hematology analyzer (Nihon Kohden Corp., Shinjuku-ku, Tokyo, Japan) and the other was processed with EDTA-2K for 2–3 h at room temperature and serum was obtained by centrifugation.

### Statistical Analysis

Statistic analysis was performed using One-Way ANOVA in Statistical Product and Service Solutions (SPSS V 19.0, IBM Corp., New York, USA). When equal variances were assumed after homogeneity of variance test, the Tukey multiple comparisons test was used. When equal variances were not assumed after homogeneity of variance test, Tamhane’s T2 multiple comparisons test was used. Differences were considered statistically significant at p < 0.05.

## Results and Discussions

### Synthesis and Characterization of F-PEG-Chol

The ^1^H-NMR spectra was the same as the previous paper^26^, which proved that F-PEG-Chol was successfully synthesized by the modified two-step reaction. Due to the optimized synthetic process parameters, the purification of the intermediate H_2_N-PEG-Chol became dramatically simpler than before^25^, thereby improving the efficiency and yield.

### Preparation and Characterization of FLP/PEDF

**Figure 2:**
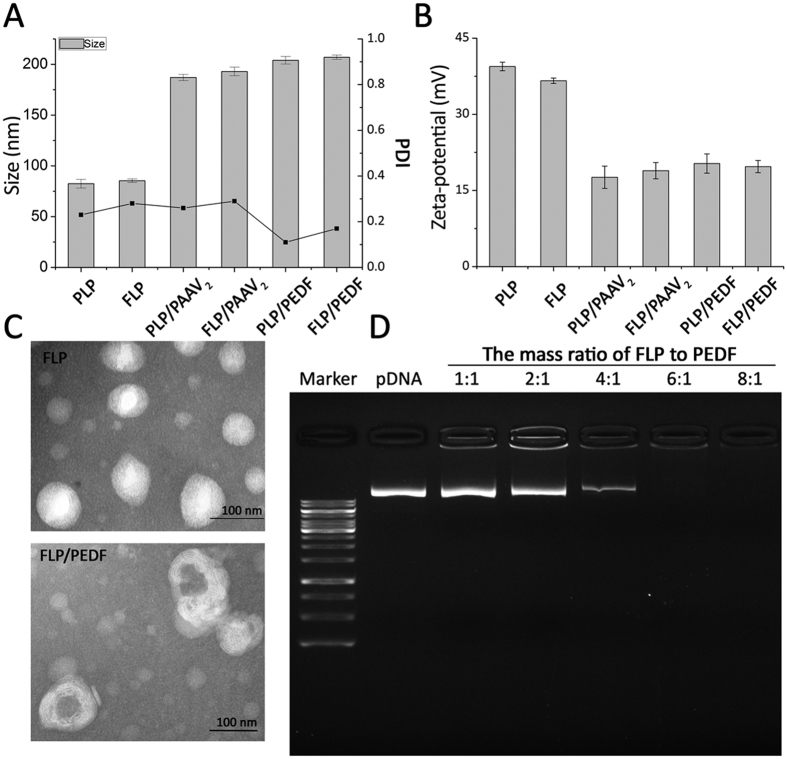
Pharmaceutical characteristics of FLP/PEDF by comparable study. (**A**) Mean diameters and polydispersity index (PDI) of nano-liposomes and nano-lipoplexes. Mean ± SD, n = 3. (**B**) Zeta-potentials of nano-liposomes and nano-lipoplexes. Mean ± SD, n = 3. (**C**) Morphological profiles of FLP and FLP/PEDF by transmission electron microscope (TEM). (**D**) Agarose gel electrophoresis was used to test the gene loading capacity of FLP. When the mass ratio of FLP to PEDF gene was up to 6:1, PEDF gene was completely encapsulated into FLP.

FLP was successfully prepared by a simple self-assembly method. As shown in Fig. 2A,B, the mean particle size of FLP was about 80 nm with a narrow polydispersity index (PDI) and the zeta-potential of FLP was around 35 mV. After incubation with PEDF gene to form FLP/PEDF, the diameter increased to about 200 nm while the zeta-potential decreased to around 20 mV due to the electrostatic interaction between gene and cationic nano-liposomes. When plasmid DNA with a negative charge bound with liposomes, the positive charge of liposomes was neutralized, which led to a decrease in the positive charge of the formed lipoplex. Besides, the sizes of lipoplexes increased mainly resulted from the plasmid DNA incubated on the surface to form a hydration shell. PLP and PLP/PEDF also had similar results in the diameter and zeta-potential. Figure 2C displayed the morphological characteristic of FLP/PEDF by TEM as compared to FLP. FLP/PEDF, presenting a typical lipid-membrane structure with spherical outline, conforming to the measurement by dynamic light scattering.

Agarose gel electrophoresis could analyze the gene loading of FLP because the free PEDF gene un-entrapped into nano-liposomes could be detected and shown as a bright band with the same electrophoresis mobility shift as pDNA. As presented in Fig. 2D, the free PEDF gene decreased as the FLP amount increased. When the mass ratio of FLP to PEDF gene was up to 6:1, PEDF gene was completely encapsulated into FLP, which means if the mass ratio was no less than 6:1, PEDF could be encapsulated into FLP completely such as 8:1 and even higher. It could be inferred that FLP were one of promising vectors with high capacity for PEDF gene delivery.

### HeLa cells Overexpressed FRα

**Figure 3:**
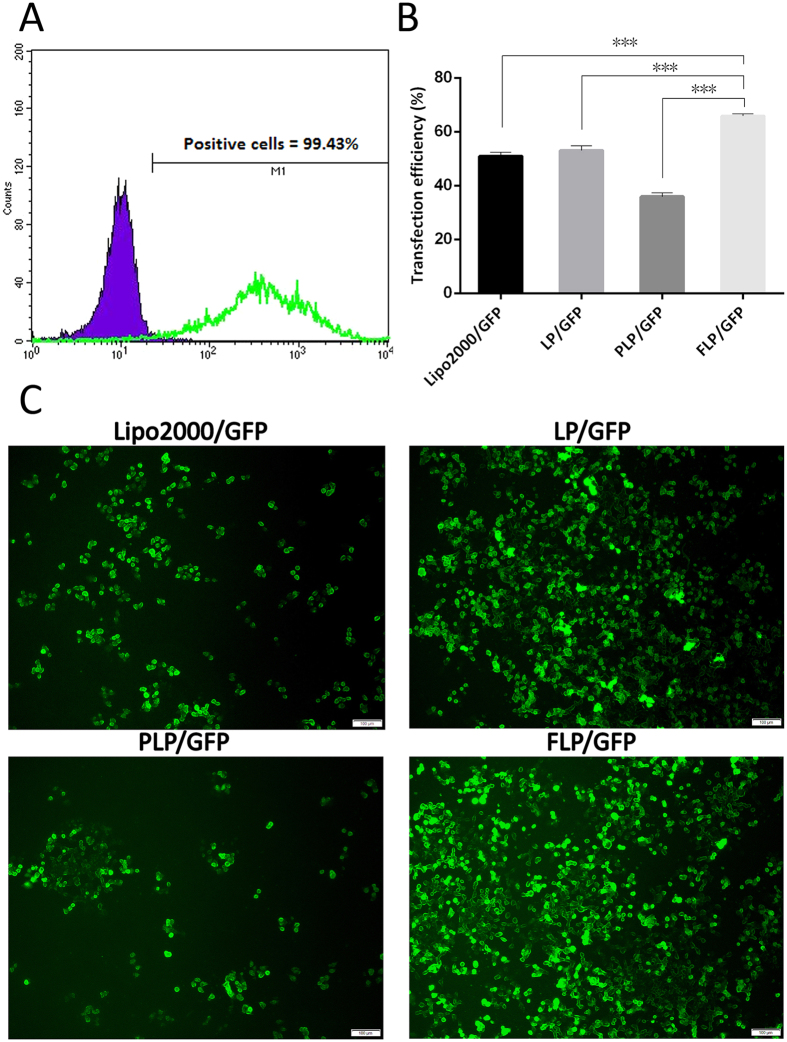
Detection of FRα expressed on human cervical cancer cells (HeLa cells) and transfection assessment of FLP/GFP in HeLa cells. (**A**) 99.43% of HeLa cells express FRα. (**B**) Transfection efficiency was measured by flow cytometer in nano-lipoplexes treated cells. FLP achieved the most efficient expression of GFP (green fluorescent stains). Mean ± SD, n = 3, ***p < 0.001. (**C**) The representative images of transfected HeLa cells by inverted fluorescence microscope.

The receptor detection assay demonstrated that 99.43% HeLa cells expressed FRα (Fig. 3A), indicating that HeLa cells we obtained were suitable model cells to investigate the targeting therapeutic effect of FLP/PEDF mediated by FRα.

### FLP Transfected HeLa cells with High Efficiency

The pGFP, a tool gene, was selected to assess the transfection efficiency of FLP. As seen in Fig. 3B,C, GFP achieved the most efficient expression in HeLa cells treated by FLP/GFP among the four groups. The transfection efficiency of FLP/GFP was significantly higher than PLP/GFP (p < 0.001), illustrating that the cells had enhanced uptake of FLP/GFP by their FRα. PEGylation is thought to decline the cell uptake efficiency^31^. Actually, PLP/GFP presented lower transfection ability than LP/GFP in this study (p < 0.001). However, FLP/GFP make the negative effect of PEGylation negligible through introduction of folate ligand. Lipo2000 is one of commercial transfection reagents used widely because of its high transfection efficiency^32^,^33^. FLP/GFP exhibited higher transfection efficiency than Lipo2000 in HeLa cells (p < 0.001), indicating that FLP were potential gene vectors for cervix cancer therapy.

### FLP/PEDF Inhibited the Growth of HeLa Cells and HUVEC Cells *in vitro*

**Figure 4:**
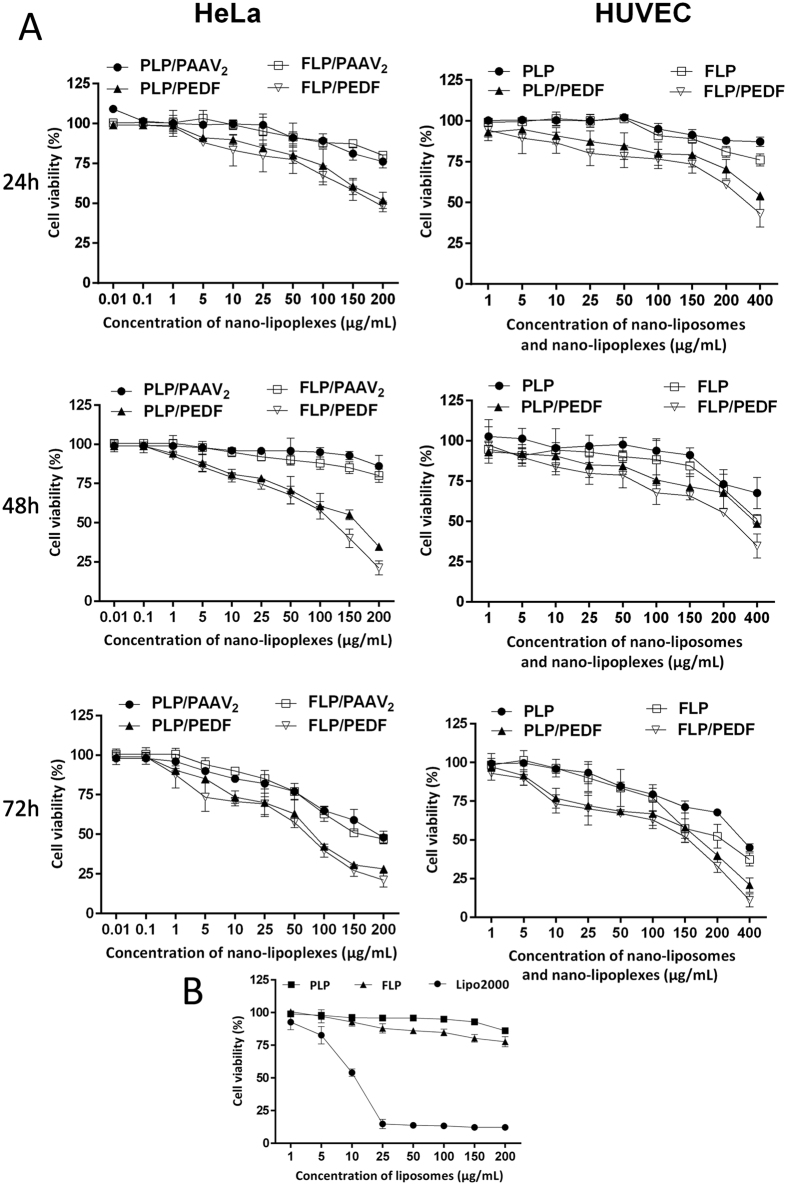
The cytotoxic study by MTT assay. Mean ± SD, n = 3. (**A**) The toxicity of different nano-lipoplexes on HeLa cells and the growth inhibition effect of nano-liposomes and nano-lipoplexes on human umbilical vein endothelial cells (HUVEC Cells) after co-culture for 24 h, 48 h and 72 h, which were concentration-dependent and time-dependent. (**B**) The toxicity of Lipo2000, PLP and FLP on HeLa cells after 48 h of treatment.

The cytotoxicity of FLP/PEDF on HeLa cells and HUVEC cells was investigated using FLP/PAAV_2_ and PLP/PEDF as the main contrast agents. The results demonstrated that the growth inhibition effects of FLP/PEDF on HeLa cells and HUVEC cells were concentration-dependent and time-dependent (Fig. 4A). FLP/PAAV_2_ were cytotoxic to HeLa cells, probably because of the positive surface charge^34^. FLP/PEDF inhibited the growth of HeLa cells more efficiently than FLP/PAAV_2_, implicating the expressing PEDF had better *in vitro* activity. More strongly cytotoxic effects of FLP/PEDF on HeLa cells were found when compared to PLP/PEDF, which might be attributed to the enhanced expression of PEDF mediated by FRα expressed in HeLa cells. We previously reported that PLGA nanoparticles loaded with PEDF gene could dramatically inhibit HUVEC cells growth and antiangiogenesis^16^,^17^. FLP/PEDF developed in this study also had similar effect. Lipo2000 only has limited application due to its toxicity. FLP had markedly lower cytotoxicity than Lipo2000 (Fig. 4B) probably because of the reduced zeta potential ascribing to PEGylation. All the data indicated that FLP with low toxicity was suitable to carry PEDF and FLP/PEDF might have the potential to inhibit the tumor growth and partly angiogenesis *in vitro*.

### FLP/PEDF Inhibited Adhension, Invasion and Migration of HeLa cells *in vitro*

**Figure 5:**
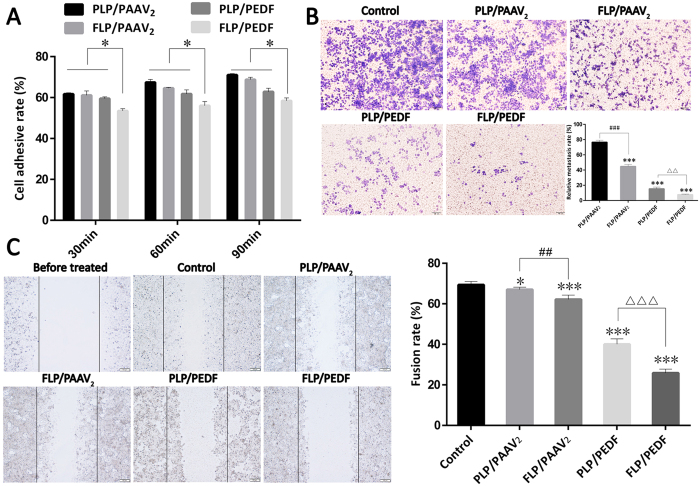
*In vitro* anti-metastasis research of FLP/PEDF. Mean ± SD, n = 3. (**A**) Adhesion assay. FLP/PEDF significantly decreased HeLa cells adhesive rate at all the determined time points compared to control, PLP/PEDF, FLP/PAAV_2_ and PLP/PAAV_2_. *p < 0.05. (**B**) Representative images of migrated HeLa cells derived from invasion assay and relative metastasis rates of HeLa cells. FLP/PEDF observably restrain HeLa cells invasion. ***p < 0.001, all the nano-lipoplexes versus control. ^△△^p < 0.01. ^###^p < 0.001. (**C**) Representative images of scratch assay and fusion rates of HeLa cells. *p < 0.05, PLP/PAAV_2_ versus control. ***p < 0.001, the other three nano-lipoplexes versus control. ^##^p < 0.01. ^△△△^p < 0.001.

PEDF has been reported to play an important role on the metastasis of tumor cells^35^,^36^. It could suppress lung and bone metastasis of osteosarcoma^37^, reducing the transfer of melanoma to lung and liver^38^. Therefore, the effect of FLP/PEDF on HeLa cells metastasis *in vitro* was investigated through the adhesion, invasion and migration assay. As shown in Fig. 5A, the adhesion rate of HeLa cells rose over time in all the four groups treated by nano-lipoplexes, among which cells transfected by FLP/PEDF had the lowest adhesion rate at all the determined time points (p < 0.05). FLP/PEDF reduced the adhesion of HeLa cells more significantly than PLP/PEDF, probably attributing to the enhanced expression of PEDF. The invasion and scratch assay also revealed the similar trend among the four nano-lipoplexes: FLP/PEDF presented the strongest inhibition of HeLa cells invasion and the lowest cell fusion rate (Fig. 5B,C, respectively). According to the data, it could be inferred that FLP/PEDF could inhibit the metastasis of HeLa cells by affecting their adhesion, invasion and migration.

### FLP/PEDF Inhibited the Growth of Abdominal Metastatic Tumor from Cervical Cancer *in vivo*

**Figure 6:**
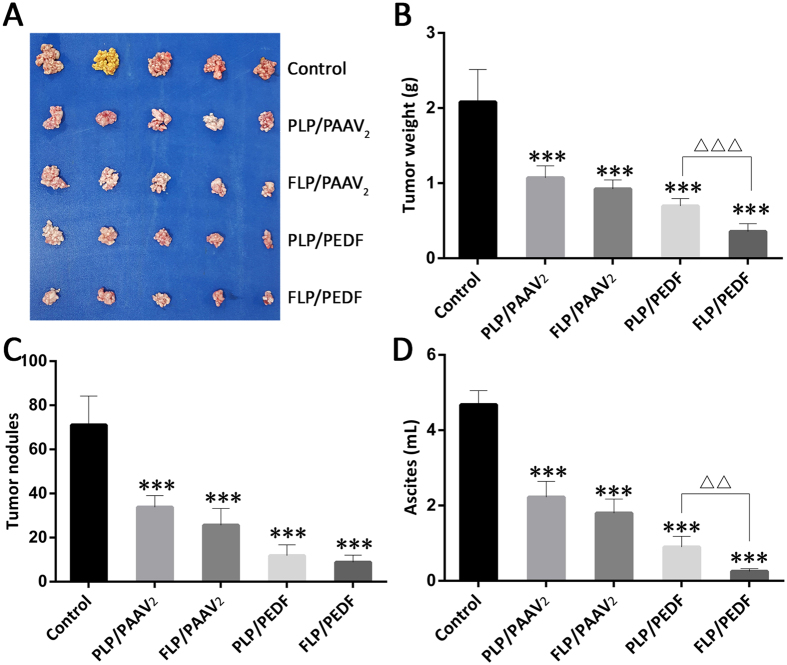
*In vivo* anti-tumor effect of FLP/PEDF by comparison with control, PLP/PAAV_2_, FLP/PAAV_2_, PLP/PEDF. The abdominal metastatic tumor model of cervical cancer was established by injecting HeLa cells into the abdomen, and these nude mice were treated by intraperitoneal injection. FLP/PEDF exhibited a superior anti-tumor effect. Mean ± SD, n = 5. ***p < 0.001, all the nano-lipoplexes versus control. (**A**) Photographs of cervical tumor tissues. (**B**) Weight of tumors. ^△△△^p < 0.001. (**C**) Amount of tumor nodules. (**D**) Volume of ascites. ^△△^p < 0.01.

The abdominal metastatic tumor model of cervical cancer was successfully established by injecting HeLa cells into the abdomen. The intraperitoneal injection for FLP/PEDF administration was selected in this study, which would achieve higher anti-tumor efficacy and be easy to bring about the clinic transformation. The therapeutic results displayed that FLP/PEDF could more efficiently diminish tumor nodules (Fig. 6A,B), decrease tumor weight (Fig. 6C) and reduce the formation of relative ascites (Fig. 6D) than all the contrast preparations. The folate ligand in FLP/PEDF might selectively deliver the nano-lipoplexes to HeLa cells by the specific recoganization of FRα having high affinity with folic acid^39^. Furthermore, the enhanced uptake of FLP/PEDF mediated by FRα probably contributed to the improvement of PEDF expression, hence leading to stronger anti-tumor activity than PLP/PEDF. Interestingly, the nano-lipoplexes encapsulating PAAV_2_ including FLP/PAAV_2_ and PLP/PAAV_2_ also had tumor growth inhibition effect. This phenomenon might result from the cytotoxic effect of vectors with positive surface charge themselves and the related humoral immune reaction stimulated by the plasmid vector itself. Thus, FLP/PEDF with multiple effects could play more forceful anti-cancer effect on cervical cancer *in vivo* than single PEDF treatment^13^.

### FLP/PEDF Up-regulated the PEDF Expression in Tumor Tissues and Serum

**Figure 7:**
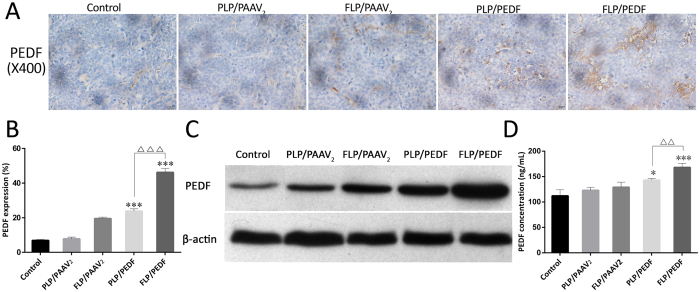
FLP/PEDF dramatically up-regulated the PEDF expression in tumor tissues and serum. (**A**) Representative immunohistochemical image of PEDF (brown stains) expressed in tumor tissues in each group (×200). (**B**) Statistical results of PEDF in tumor tissues. ***p < 0.001, FLP/PEDF and PLP/PEDF versus control. ^△△△^p < 0.001. (**C**) Semi-quantitative assay of PEDF expressed in tumor tissues by Western blot analysis. (**D**) Concentrations of PEDF secreted into serum by ELISA assay. ***p < 0.001, FLP/PEDF versus control. *p < 0.05, PLP/PEDF versus control. ^△△^p < 0.01.

The PEDF level in cervix carcinoma was reported to be lower than that in normal cervical tissue^40^. Introduction of exogenous PEDF could enhance the therapeutic efficacy of cervical cancer. IHC staining and western blot analysis were employed to detect the expression of PEDF. As shown in Fig. 7A,B, PEDF (brown stain) expressed in all tumor tissues from the five groups. Only extremely low PEDF expression was found in the control group, while a large amount of PEDF dispersed in the tumor tissue of FLP/PEDF treated group. FLP/PEDF resulted in a significant increase of near 100% in PEDF expression in HeLa cells *in vivo* as compared to PLP/PEDF (p < 0.001), which might be caused by the modification of folate ligand on the nano-lipoplexes as discussed above. The western blot result displayed in Fig. 7C further confirmed the data provided by IHC: FLP/PEDF indeed enhanced the PEDF expression dramatically in the abdominal metastatic tumor from cervical cancer. PEDF, a typical secretory protein, could excrete into blood except tissue distribution^41^. Thus, the ELISA analysis was carried out to determine the PEDF concentration in mice serum. As seen in Fig. 7D, all the tumor bearing mice had PEDF detected in serum. The serum PEDF slightly elevated after treated by PLP/PEDF (p < 0.05), while dramatically increased after treatment with FLP/PEDF. Taken together, FLP/PEDF could effectively transfect HeLa cells *in vivo* and successfully achieved PEDF expression with high efficiency, demonstrating that FLP were hopeful vectors for PEDF delivery specific to cervix cancer cells *in vivo* by intraperitoneal administration.

### FLP/PEDF Suppressed Angiogenesis, Inhibited Cell Proliferation and Induced Cell Apoptosis *in vivo*

**Figure 8:**
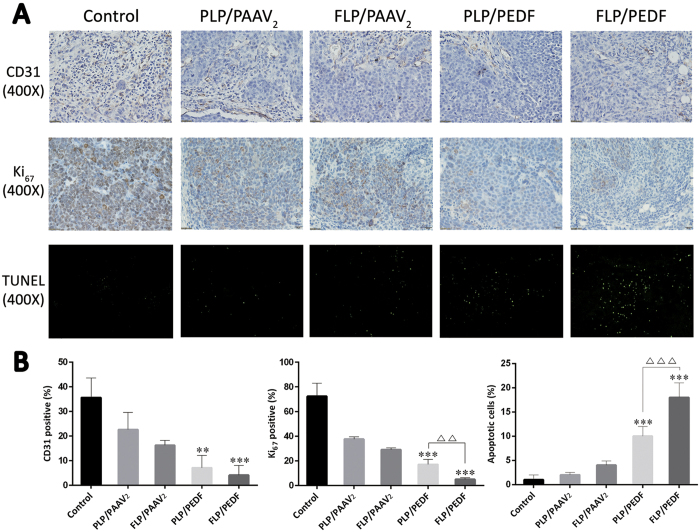
FLP/PEDF remarkably suppressed angiogenesis, inhibited cell proliferation and induced cell apoptosis *in vivo*. The microvessel density (MVD) was assessed by CD31 staining. The proliferative profile of HeLa cells *in vivo* was investigated through Ki_67_ staining analysis. Moreover, TUNEL was employed to evaluate tumor cell apoptosis. (**A**) Representative immunohistochemical images of tumor sections in each group (×400). (**B**) Statistical results of MVD, proliferative cells and apoptotic cells. **p < 0.01, treated group versus control. ***p < 0.001, treated group versus control. ^△△^p < 0.01. ^△△△^p < 0.001.

The potential mechanism underlying the anti-tumor efficacy of FLP/PEDF-based therapy was investigated by CD31 staining, Ki_67_ staining and TUNEL assay. It had been reported that cervical cancer was angiogenesis-dependent^36^. PEDF in an increasing level resulted in tumor suppression by inhibiting neovascularization^37^. The endothelial cells of tumor new blood vessels express CD31, which is often used to evaluate the degree of tumor angiogenesis and imply a rapidly growing tumor. Thus, CD31 expression was examined by IHC staining to study the effect of FLP/PEDF on angiogenesis in this study. As shown in Fig. 8A,B, FLP/PEDF sharply reduced CD31 positive cells (brown stains) to 5% and significantly decreased microvessel density (MVD) compared with PLP/PEDF and control (p < 0.001). This mainly ascribed to the improved expression of PEDF. PEDF was reported to suppress tumor cell proliferation^13^. The Ki_67_ staining analysis (brown stains) in this study showed that FLP/PEDF treatment resulted in 10% of Ki_67_ expression, whereas PLP/PEDF treatment resulted in 20% of Ki_67_ expression. FLP/PEDF, with elevated PEDF expression, presented significantly efficient inhibition activity on the proliferation of HeLa cells *in vivo* than PLP/PEDF (p < 0.01). Furthermore, TUNEL was used to evaluate tumor cell apoptosis in the mice treated by the four nano-lipoplexes because it could detect DNA fragmentation derived from apoptotic signals. According to the green fluorescent spots, the tumor cell apoptosis was specifically increased (18-fold, versus control, p < 0.001) when the mice were treated by FLP/PEDF but only 10-fold after they were exposed to PLP/PEDF. Similar to the suppressive effect of tumor growth, FLP/PAAV_2_ and PLP/PAAV_2_ also slightly affected angiogenesis of tumor, proliferation and apoptosis of HeLa cells. Both nano-lipoplexes with positive zeta potentials might be cytotoxic to the endothelial cells in tumor tissue, which caused down-regulated neovascularization. Moreover, they were capable of inhibiting proliferation and promoting apoptosis *in vivo* which was similar to the cytotoxic effect *in vitro*. These results demonstrated that the highly efficient anti-tumor activity of FLP/PEDF might involve the suppression of angiogenesis, inhibition of cell proliferation and cell apoptosis by not only PEDF with enhanced expression but the vector itself.

### FLP/PEDF were Relatively Non-toxic to Female Mice Bearing Tumor

**Figure 9:**
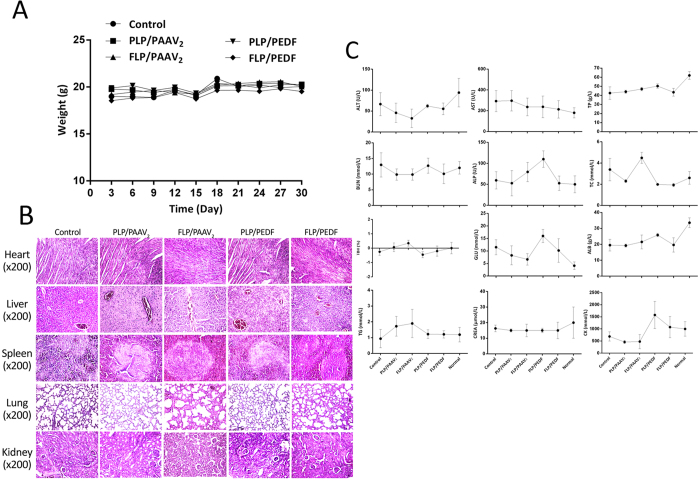
Preliminary safety evaluation of FLP/PEDF in female mice bearing tumor. Mean ± SD, n = 5.(**A**) Body weight of tumor-bearing mice in each group. FLP/PEDF caused no weight change during treatment. (**B**) Serological and biochemical indexes determined at the therapeutic end point. FLP/PEDF treatment contributed to better AST, TC and CK levels approaching to the normal ranges. (**C**) Representative H&E images (×200) of vital organs including heart, liver, spleen, lung and kidney. FLP/PEDF had no obvious toxicity on these tissues.

PEDF was reported to have no tissue specificity^39^, which might cause side effects on normal endothelial cells or some normal tissues. Our ELISA analysis also confirmed that PEDF indeed secreted into blood, although FLP/PEDF were administered by locally intraperitoneal injection. Thus, the preliminary safety evaluation of FLP/PEDF was performed in the female mice bearing tumor by body-weight monitoring during treatment, organs biopsy, serological and biochemical analysis at the therapeutic end point. In the whole process of animal experiments, the appearance of mice remained hairless and albino in background until they were sacrificed. Normal urine and fecal appearances were found during treatment in all the groups. Near the end of treatment, the nude mice were unable to move freely because of malignancy-related ascites especially in control group but not in FLP/PEDF-treated one. The total body weight of FLP/PEDF-treated mice had no difference with those treated by other nano-lipoplexes or control (Fig. 9A). HE staining presented no significantly toxic pathological changes in the heart, liver, spleen, lung and kidney derived from FLP/PEDF-treated mice as shown in Fig. 9B. Moreover, all the biochemical indexes illustrated that the vital organs’ functions of FLP/PEDF-treated mice were present at the similar levels according to the data of normal mice (Fig. 9C), among which AST, TC and CK were approaching to the normal ranges. It could be inferred that FLP/PEDF were one of relatively safe formulations by intraperitoneal administration for the treatment of female mice bearing tumor probably because the increased PEDF in blood is not enough to induce toxic effects.

## Conclusion

The targeting lipid F-PEG-Chol was expectably synthesized by a novel method, and FRα-targeted nano-lipoplexes loaded with PEDF gene (FLP/PEDF) were successfully developed by a film dispersion method for the first time. The cervical cancer therapeutic effects were systemically investigated *in vitro* and *in vivo*. FLP/PEDF could efficiently transfect HeLa cells, inhibit the growth of HeLa cells and HUVEC cells, suppress adhesion, invasion and migration of HeLa cells *in vitro*. Moreover, FLP/PEDF exhibited a superior anti-tumor effect than non-targeting nano-lipoplexes, possibly due to selective delivery of PEDF to HeLa cells involving the over-expressing FRα with specific recognization of folate ligand. FLP/PEDF play forceful effect on cervical cancer treatment by not only PEDF at an elevating level but the vector itself (involving the nano-liposomes with positive surface charge and plasmid vector). The deep study of the mechanism demonstrated that the highly efficient anti-tumor activity of FLP/PEDF was related to the suppression of angiogenesis, inhibition of cell proliferation and cell apoptosis. The preliminary safety evaluation confirmed that FLP/PEDF by intraperitoneal administration were relatively safe. In sum, FLP were promising carriers for PEDF gene and FLP/PEDF were potential for future cervical cancer gene therapy.

## Additional Information

**How to cite this article**: Yang, Y. *et al*. Promising Nanocarriers for PEDF Gene Targeting Delivery to Cervical Cancer Cells Mediated by the Over-expressing FRα. *Sci. Rep.* **6**, 32427; doi: 10.1038/srep32427 (2016).
